# Tubulin Dimers Oligomerize before Their Incorporation into Microtubules

**DOI:** 10.1371/journal.pone.0003821

**Published:** 2008-11-27

**Authors:** Julien Mozziconacci, Linda Sandblad, Malte Wachsmuth, Damian Brunner, Eric Karsenti

**Affiliations:** 1 Pierre et Marie Curie University, UMR 7600 LPTMC, Paris, France; 2 EMBL, Cell Biology and Biophysics Unit, Heidelberg, Germany; Wellcome Trust Sanger Institute, United Kingdom

## Abstract

In the presence of GTP, purified dimers of α- and β-tubulin will interact longitudinally and laterally to self-assemble into microtubules (MTs). This property provides a powerful *in vitro* experimental system to describe MT dynamic behavior at the micrometer scale and to study effects and functioning of a large variety of microtubule associated proteins (MAPs). Despite the plethora of such data produced, the molecular mechanisms of MT assembly remain disputed. Electron microscopy (EM) studies suggested that tubulin dimers interact longitudinally to form short oligomers which form a tube by lateral interaction and which contribute to MT elongation. This idea is however challenged: Based on estimated association constants it was proposed that single dimers represent the major fraction of free tubulin. This view was recently supported by measurements suggesting that MTs elongate by addition of single tubulin dimers. To solve this discrepancy, we performed a direct measurement of the longitudinal interaction energy for tubulin dimers. We quantified the size distribution of tubulin oligomers using EM and fluorescence correlation spectroscopy (FCS). From the distribution we derived the longitudinal interaction energy in the presence of GDP and the non-hydrolysable GTP analog GMPCPP. Our data suggest that MT elongation and nucleation involves interactions of short tubulin oligomers rather than dimers. Our approach provides a solid experimental framework to better understand the role of MAPs in MT nucleation and growth.

## Introduction

Microtubules (MTs) are tubular structures resulting from the assembly of α- and β-tubulin-dimers. They constitute an important part of the cellular scaffold, providing a network of tracks for intracellular transport and for separating chromosomes during mitosis. Under appropriate conditions and in the presence of the nucleotide GTP, purified tubulin dimers self assemble into MTs [1]. The structure of tubulin dimers is known at atomic resolution [2] and their arrangement inside the MT wall has been precisely described by 3D reconstruction after electron microscopy imaging [3]. Dimers are interacting longitudinally in protofilaments, which form the MT wall through lateral interactions. In vitro, a sheet like structure of protofilaments is present at the tip of growing MTs, suggesting that microtubules grow through the incorporation of tubulin molecules into this sheet that then closes into a tube [4]. Whilst much is now known about MT dynamic behavior at the micrometer scale, the mechanism behind their assembly at the molecular level remains unclear. Two models of spontaneous MT nucleation have been proposed [5]. One posits that tubulin-dimers first interact longitudinally to form protofilaments that subsequently interact laterally to form a tube. The second proposes that lateral tubulin-dimer interactions form a closed ring, which serves as a template on which subunits are longitudinally added. MT elongation at molecular resolution is also a subject of recent controversy. Some authors posit that tubulin dimers are forming short longitudinal oligomers that are subsequently incorporated at the MT ends [6] whereas others claim that MTs are elongating through incorporation of single dimers [7]. In order to obtain a firmer ground on which to understand how microtubule assembly occurs at the molecular scale, we set out to determine the equilibrium size of tubulin oligomers at very low tubulin concentration. This was achieved using two independent methods: quantitative analysis of EM-resolved tubulin oligomers and FC(C)S analysis of a two-color labeled tubulin mix.

Fitting the experimental data to a theoretical model gave us a precise estimate of the longitudinal binding energie (E_long_) of GMPCPP (a very slow hydrolysable GTP analog) and GDP tubulin-dimers. Knowing *E*_long_ we were able to estimate the length of tubulin oligomers under conditions of MT self assembly. Our results fully support a model in which short protofilament intermediates are central to MT nucleation and elongation. This has important implications for the understanding of the effects of MT-associated proteins on microtubule dynamics under well defined experimental conditions.

## Results

### Short tubulin protofilaments form prior to MT assembly

**Figure 1:**
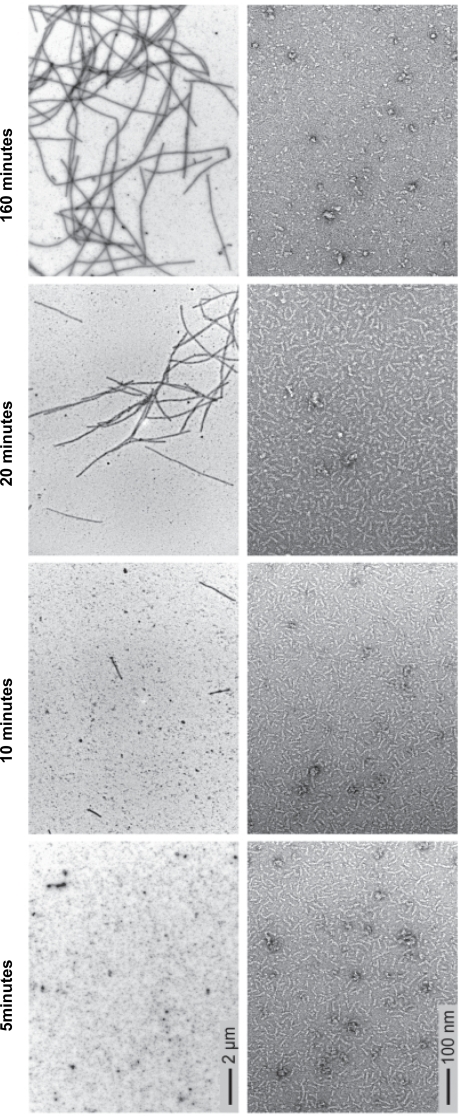
MT nucleation at 2.5 µM tubulin with GMPCPP. Electron microscopy images of four consecutive time points during microtubule nucleation and elongation (5, 10, 20 and 160 minutes). Two different magnifications were used in order to monitor microtubule growth and tubulin oligomer quantity.

To address the question whether or not tubulin dimers preferentially pre-assemble into oligomers before being incorporated into MTs during nucleation and growth we initiated a quantitative analysis of the different stages of MT self-assembly using EM. We first tried to follow tubulin assembly on EM grids that were negatively stained at various time points following the addition of 1 mM GTP to a solution containing 25 µM tubulin. Under these conditions the formation of tubulin oligomers, mostly with filamentous appearance but also globular, clearly preceded the appearance of MTs (Figure S1). Unfortunately, a quantitative analysis was impossible because of the high density of structures, which resulted in a low signal to noise ratio. To decrease the density of structures we replaced GTP with GMPCPP, a non-hydrolysable GTP analog that enables MT nucleation at much lower tubulin concentrations [8]. Another advantage of the lack of hydrolysis is that no destabilization occurs allowing observation of the initial interaction steps in the complete absence of disassembling structures and under conditions satisfying the law of mass action. Immediately after adding 1 mM GMPCPP to a 2.5 µM tubulin solution oligomers, similar to those observed in the GTP preparations, became visible (Figure 1). After 5 minutes, the first MTs appeared whilst the density of tubulin oligomers remained constant. At later time points the density of these structures decreased while MT number and length continued to increase. After 160 minutes, MTs were very long and especially the filamentous oligomer structures had almost completely disappeared, probably as a result of the incorporation into MTs.

**Figure 2:**
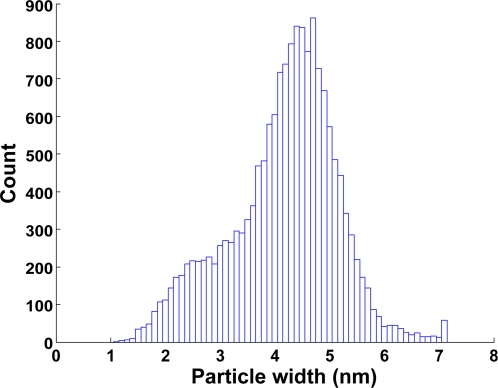
Tubulin oligomer width. The width distribution of particles formed at 0.5 µM tubulin concentration with GMPCPP is shown.

**Figure 3:**
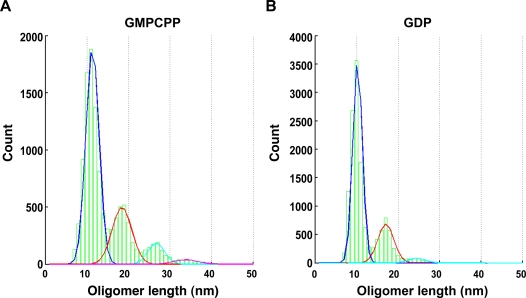
Tubulin oligomer length. The equilibrium length distribution of tubulin oligomers formed at 0.5 µM tubulin concentration. Green boxes show the actual distribution. The four Gaussians fitting the distribution are shown in blue, red, cyan and pink respectively. (A) Distribution in the presence of GMPCPP. (B) Distribution in the presence of GDP.

To determine whether the observed filamentous oligomers corresponded to protofilaments, we performed a large-scale image analysis and monitored the distribution of the filament sizes. This was done using 0.5 µM tubulin, which is just below the concentration needed for GMPCPP-induced MT nucleation. Filament sizes were measured at seven consecutive time points (from 1 minute to several hours) after initiating the process by GMPCPP addition. The length distribution of the filaments did not vary over time, indicating that equilibrium was reached very rapidly. Figure 2 shows the distribution of filament width, which fits a bell-shaped curve centered on 4.5 nm. This matched well with the width of tubulin dimers. Smaller particles were also present, which are most likely caused by various nonspecific objects present on the grid. We next measured the lengths of those filaments having a width lying inside one standard deviation from the 4.5 nm mean. Using a multi-Gaussian fitting algorithm we found that their lengths were distributed over four Gaussians with mean values centered on 10, 18, 26 and 34 nm (Figure 3). The 8 nm periodicity matches the longitudinal size of tubulin dimers. The 2 nm aberration of the peaks from the known length of the respective tubulin dimers or multimers, must be due to the negative staining procedure, which makes objects appear slightly bigger. We conclude that the filaments observed were indeed short MT protofilaments. The measured oligomer length distribution yields an average of 1.6±0.1 tubulin dimers per oligomer and reveals that only 40%±5% of the tubulin exists as single dimers (Figure 3). Also when repeating the experiment in the presence of GDP instead of GMPCPP we found that no more than 60%±5% of the tubulin exists in the form of a single dimer, corresponding to a mean length of 1.3±0.1 dimers per oligomer (Figure 3). This shows that tubulin has an intrinsic ability to form oligomers independent of the bound nucleotide, although the nucleotide does influence this capacity.

**Figure 4:**
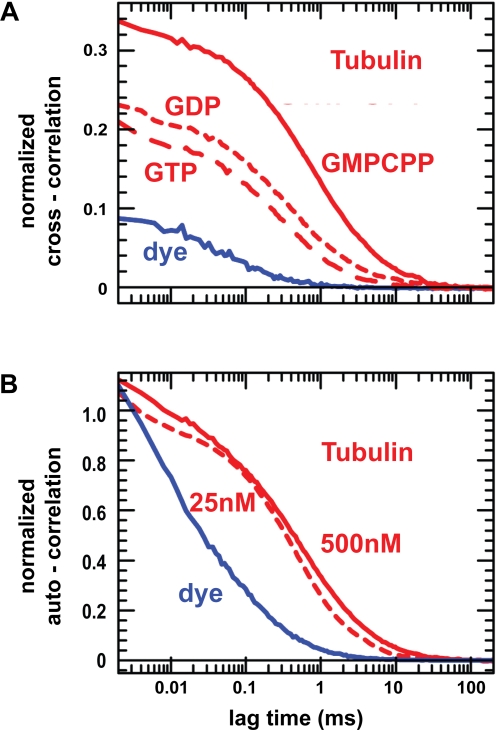
Fluorescence correlation spectroscopy analysis. (A) Cross-correlation functions of the mix of free fluorophores (solid blue curve) and of the mix of differently labeled tubulins in the presence of GMPCPP (solid red curve), GDP (short-dashed red curve), and GTP (long-dashed curve) normalized as described in Supplementary Material S1. For the tubulin mixes, we obtained amplitudes of ratioG = 0.33 (GMPCPP), 0.22 (GDP), and 0.20 (GTP; see Eq. S4) well above the baseline value of 0.09. (B) Measured and normalized autocorrelation functions of free Cy5 fluorophore (solid blue curve) and Cy5-labeled tubulin at 25 nM (dashed red curve) and 500 nM (solid red curve). For tubulin at 500 nM, a diffusion coefficient of *D*_tubulin_ = 23.4±2.2 µm^2^s^−1^ was found. This is 2-fold smaller than for the bona fide free tubulin dimers measured at 25 nM due to oligormer formation.

In our EM analysis, tubulin oligomers were measured after being adsorbed to a carbon surface, which could in principle catalyze oligomer formation. To exclude this possibility, we set out to determine the oligomerization state in solution using fluorescence cross-correlation spectroscopy (FCCS) experiments. Labeled tubulin was prepared with the two different fluorophores Cy3 and Cy5. Labeling did not impede oligomer formation on the EM grids (Figure S2). We first recorded fluorescence intensity fluctuations for an equimolar mix of the free fluorophores (0.5 µM total concentration). The resulting measurements were used as a reference, representing non-interacting particles (Figure 4A). We then repeated the measurements using an equimolar mix of both labeled tubulins (0.5 µM total concentration) in the presence either of GDP, GTP or GMPCPP. The amplitudes of the resulting cross-correlation functions were strongly increased as compared to the reference suggesting that a significant fraction of the tubulin dimers was in an oligomeric state (Figure 4A). Confirming our measurements on EM grids, GMPCPP-tubulin oligomerized better than GDP-bound tubulin. Surprisingly, the oligomerization state of GTP-bound tubulin was virtually identical to that of the GDP-bound form suggesting that unlike GMPCPP, GTP may not at all influence longitudinal tubulin interactions and that its main role is to increase the lateral interaction energy of tubulin.

We then used the autocorrelation function of the Cy5-labeled GDP-tubulin at 25 nM to calculate the diffusion coefficient of the free tubulin. The obtained value of 47.5 µm^2^/s was in good agreement with the previously published 45 µm^2^/s [9]. Comparing this with the diffusion coefficient of GTP- and GMPCPP-tubulin oligomers at 0.5 µM concentration allowed us to determine the average number of tubulin dimers per oligomer in a solution containing 0.5 µM tubulin (Fig. 4B, see Supplementary Material S1). Considering a labeling density of ∼1.5 fluorophores per tubulin dimer and an exponential distribution for oligomer sizes we obtained values of 1.7±0.2 and 1.3±0.2 for GMPCPP and GTP supplemented medium respectively. Taken together our results show that, independent of their nucleotide binding state, the majority of tubulin dimers in solution assemble to form short protofilaments.

### Derivation of the longitudinal dimer interaction energy

**Figure 5:**
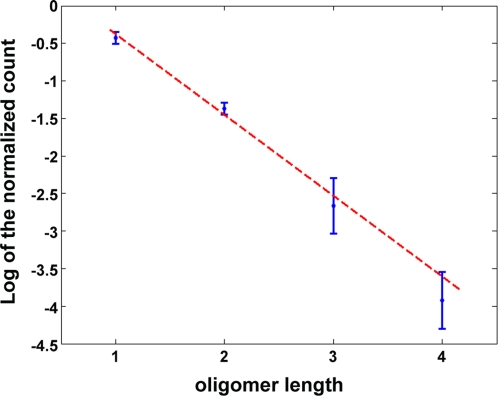
Tubulin oligomer length analysis. Fitting of the experimental results with the model. The blue points and error bars were obtained from 5 independent experiments. The red dotted line shows the best fit weighted using error bars.

The above results clearly suggest that MT assembly starts with the formation of protofilaments made of longitudinally bound tubulin dimers. It is known from macromolecular chemistry [10] that the equilibrium size distribution of a polymer formed by addition of monomers is exponential [11]. Oosawa extended this result to oligomers which are growing and shrinking from both extremities and are breaking and recombining [11]. We used this theory to derive the longitudinal binding free energy *E*_long_ from our measurements (see Supplementary Material S1). Figure 5 shows the logarithm of the tubulin oligomer length distribution that was measured in our EM experiments with GMPCPP, and that can be fitted by a straight line. Its slope provides *E*_long_. We found a best fit for *E*_long = _−14.4 kT±0.2 kT. The corresponding equilibrium constant (*K*_long_ = 3.6·10^6^ M^−1^) was now calculated taking the exponential of the free energies. A higher value of −13.6 kT±0.2 kT was extracted in preparations containing GDP instead of GMPCPP (Figure S3) in agreement with the observed shortening of tubulin oligomers.

### Visualization of lateral interactions between protofilaments

**Figure 6:**
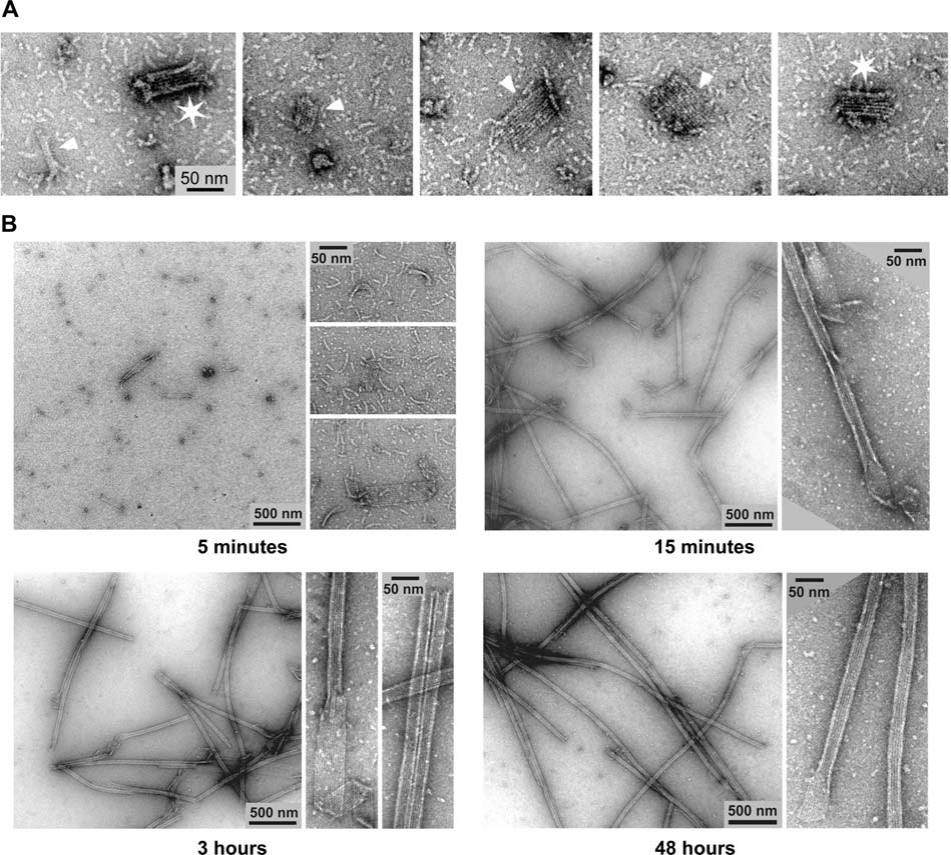
Microtubule nucleation intermediates. Images were taken at successive time points from preparations containing 2.5 µM tubulin, 1 mM GMPCPP, 10% DMSO. (A) A selection of MT intermediates as found frequently at early time points. Tubulin sheets formed by laterally associated, short oligomers (arrowheads) and short MT stumps (stars) can be seen. (B) Different time windows of MT assembly.

Our results strongly support the postulated model for MT nucleation suggesting that upon addition of GTP or GMPCPP, pre-existing, short tubulin protofilaments start interacting laterally to form sheet-like MT nucleation intermediates that subsequently close into a tube. However, we were not able to detect such nucleation intermediates, because under standard experimental conditions the nucleation process happens very fast such that intermediates must be very short-lived. To address this point, we tried to find conditions under which we could observe intermediates. DMSO has been used to observe large MT sheets and to increase the number of protofilaments in the MTs [12], [13]. DMSO slows down the closure of the tube most likely because the lateral binding angle between protofilaments may change in the presence of this solvent. We tested whether this effect would sufficiently increase the lifetime of the short-lived nucleation intermediates to allow their detection. When adding DMSO with a 10% final concentration to our MT nucleating GMPCPP preparations we could now frequently observe short tubulin sheets of various widths, obviously containing variable numbers of laterally attached protofilaments at early time points (Figure 6A). Few short MT stumps were also present in the preparation. At later time-points more closed MT structures appear and the number of sheets slowly decreases (Figure 6B). This shows that under our experimental conditions MT structures that match the expected nucleation intermediates can be observed.

## Discussion

### Implications on MT elongation

**Figure 7:**
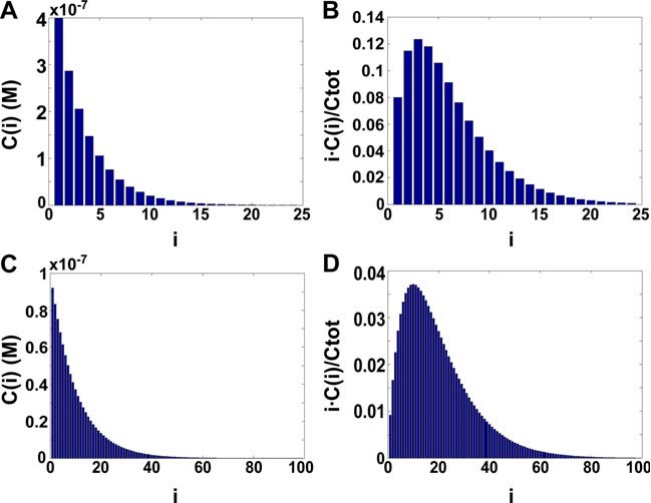
Theoretical oligomer length distribution at higher concentrations. “i” is the number of tubulin dimers per oligomer. “C(i)” is the concentration of oligomers containing “i” tubulin dimers and Ctot is the total tubulin concentration. “i•C(i)/Ctot” is therefore the fraction of dimers involved in making an oligomer of size “i”. The distributions were estimated using *E*_long_ = −14.4 kT. (A) and (B) show results obtained at 5 µM tubulin, the typical concentration used for MT assembly *in vitro*. (C) and (D) show results obtained at 10 µM tubulin concentration to mimic *in vivo* conditions and the effect of molecular crowding.

The issue of microtubule elongation at molecular scale has been addressed in two recent papers. Unexpectedly, quasi-similar experimental set-ups led the authors to very different conclusions [6], [7]. The first set of results showed that pre-formed tubulin oligomers where participating in MT elongation [6] whereas the later one argues that only single dimers are incorporated at the MT tip [7]. In this last paper the authors use the previously estimated free-energy of tubulin/tubulin interaction (in the range of −7 to −9 kT [14]–[16]) to predict that at 5 µM tubulin concentration only 5% of the tubulin dimers would be in an oligomeric form, whereas 95% of the tubulin should be in the form of individual dimers [7]. Our result for *E*_long_ is significantly lower than the previously estimated values and leads to radically different conclusions. On figure 7A we present the expected distribution of oligomers at 5 µM tubulin concentration. We find that 92% of GMPCPP-tubulin dimers will be in an oligomeric form whereas only 8% will be present as single dimers (Figure 7B). Nevertheless, the experimental results from Schek *et al*. [7] are compatible with our results assuming the following: growing MT plus ends were previously shown to form intermediate sheet structures that subsequently close into tubes [4], [17]. At the tip of the sheet single tubulin dimers would be more likely to interact longitudinally than the less motile oligomers. At the same time tubulin oligomers will participate considerably in sheet formation and MT elongation just as they do during early nucleation stages by laterally associating with longer protofilament overhangs within the sheet. Here, short tubulin protofilaments are more likely to be added than free tubulin dimers because they are able to make strong lateral interactions in addition to the longitudinal interaction. In this way both dimers and oligomers, would participate in tube elongation while the average increment of MT length could still be close to the 8 nm measured by Schek *et al* [7].

### Comparison with previously estimated values for *E*_long_

The first value reported for *E*_long_ comes from early sedimentation experiments [15]. GDP-bound tubulin dimers were shown to self-associate in the presence of magnesium and the association constant was extracted from the sedimentation profiles. The corresponding free energy value was found to be close to −9 kT. The difference to our value could be explained by a lack of GDP in the sedimentation experiments. Only 0.1 mM GDP together with up to 0.2 mM tubulin were used. At these low concentrations many of the tubulin dimers may have been lacking a bound nucleotide, which is required for proper longitudinal interaction and as a result *E_long_* would have been underestimated.

A simple model of MT nucleation from pure tubulin provided a value for *E*_long_ that was similar to those in the sedimentation experiments [14]. The model uses several free parameters, one of them being the critical tubulin concentration (Cc) above which spontaneous MT nucleation occurs. A value for Cc of 10^−5^ M was used in agreement with earlier measurements on GTP-induced MT nucleation. It was evidenced 15 years later that GTP hydrolysis slows down the early steps of MT nucleation by prohibiting the interaction between short protofilament intermediates [18]. This means that the relevant Cc to be used to estimate *E*_long_ in the model should have been that of the non-hydrolysable GMPCPP tubulin, which is around 10^−6^ M and therefore significantly lower. When doing this, we found that also in this model the resulting *E*_long_ was now close to −14 kT, which is in excellent agreement with our measurement.

Finally, Van Buren et al. [16] used simulations of MT growth to estimate *E*_long_ based on measured MT growth speeds. They estimated *E*_long_ to fall between −7 and −9 kT. Also in this model, there is one free-parameter: the association constant between two dimers *k* ^+^, which was chosen to be in agreement with the previous studies [14], [15]. Our direct measure of *E*_long_ can now be used to fix one more parameter in such theoretical studies.

From our EM images we could not estimate *E*_long_ for GTP-bound tubulin because nucleotide hydrolysis is driving the system out of equilibrium. However, two of our FCS/FCCS results suggest that for GTP-bound tubulin the *E*_long_ is similar to the one of GDP-bound tubulin. First, in FCCS the autocorrelation function is almost the same for GTP and GDP preparations. Second, the mean length of GTP oligomers obtained from FCS measurements is equal to the mean length of GDP oligomers obtained using EM. While GPMCPP mildly increases longitudinal tubulin interactions GTP does not seem to do so but rather induces nucleation by increasing lateral interactions.

### *In vivo* implications

The tubulin concentration in living cells is usually very high (up to 24 µM [19]), which should strongly favor MT assembly. Molecular crowding, induced by the high concentration of additional macromolecules in the cytoplasm, is likely to provide further enhancement [20]. We used measurements on the effect of crowding on protein oligomerization to estimate the size distribution of protofilaments before their incorporation into MTs under crowding conditions (see Supplementary Material S1). Our calculations predict that at a 10 µM tubulin concentration, linear oligomers of several hundred nm would form (Figure 7C and 7D). This would certainly impede tubulin diffusion towards MT ends and thus MT growth. Here our calculations seem incompatible with the *in vivo* situation. In cells however, a considerable number of MT-associated proteins (MAPs) are known to influence tubulin oligomerization. These may be modulating the multimerizing property of tubulin dimers. Indeed, a recent structural study of the plus end tracking proteins XMAP215, EB1 and Clip-170 suggested that those proteins share the common property of multimerizing tubulin dimers, thus acting as polymerization chaperones [21]. Alternatively, MT-destabilizing MAPs such as Stathmin, which prevents longitudinal contacts between dimers [22], could influence the length of free tubulin oligomers by tethering tubulin subunits. It is further intriguing to speculate that members of the Kinesin 8 and 13 protein families could directly act on free tubulin oligomers to reduce their length. These proteins are known to destabilize MTs presumably by exerting pulling forces on the protofilaments [23].

The experimental approach we present here not only gives detailed insights into the basic mechanisms of spontaneous MT assembly under well-defined experimental conditions, it also provides a powerful tool for the quantitative description of the mechanisms, with which MT nucleation and growth are regulated. For example, one can now use it to explore effects on MT assembly that are caused by varying defined parameters such as the source of the tubulin used or the ion concentration. It can furthermore be used to investigate the effects on MT nucleation and growth of the various MAPs, which should help to understand their role in MT assembly *in vivo*.

## Materials and Methods

### Sample preparation

Porcine tubulin was purified as described [24], and stored in BRB80 buffer (80 mM PIPES, 1 mM MgCl_2_, 1 mM EGTA, pH 6.8) at −80°C. 0.1–2.5 µM tubulin was polymerized in BRB80 buffer, supplemented with 2.5 µM MgCl_2_ and 1 mM GMPCPP or GDP. Nucleation intermediates were visualized using 25 µM bovine tubulin in BRB80, 1 mM GMPCPP, 4 mM MgCl_2_ and 10% DMSO that was subsequently diluted 10× in BRB80, MgCl_2_ and DMSO or using 2.5 µM porcine tubulin in the same buffer without dilution steps (figure 6B). All experiments were performed at room temperature and for imaging all samples were stained for EM (see below). For FCS experiments tubulin was labeled according to Peloquin *el al.* [25].

### Negative staining electron microscopy

Tubulin was absorbed for 1 minute onto glow-discharged formvar- and carbon-coated grids. The samples were stained in 1.5% uranyl acetate for 25 seconds. Images were recorded on a FEI Morgagni 268D transmission electron microscope, using a Mega-View III CCD camera. Tubulin oligomers were imaged at 110,000× magnification resulting in a pixel size of 0.53 nm.

### Image analysis

Images used for quantitative analysis were obtained after absorption of a solution containing 0.5 µM of tubulin polymerized in BRB80 buffer, supplemented with 2.5 µM MgCl_2_ and 1mM GMPCPP or GDP. Image analysis was done with ImageJ (http://rsb.info.nih.gov/ij/index.html). The pictures were smoothened using Gaussian filtering and an anisotropic diffusion algorithm. After background subtraction, a threshold was applied such that individual oligomers appeared as black particles on a white background (Figure S4). The area and the Feret diameter of those particles could then be measured. The Feret diameter of a particle is the greatest distance between two points within the particle (here reflecting protofilament length). Protofilament width was derived by dividing the particle area by its length. 30 to 50 pictures for each time point were analyzed (30,000 tubulin oligomers in total).

### FC(C)S experiments

FC(C)S experiments were carried out on a Zeiss ConfoCor2 FCS system using a C-Apochromat 40× 1.2W Korr water immersion lens. Cy3 was excited with 488 nm and detected with a 560–600 nm bandpass filter. Cy5 signal was excited with 633 nm and detected with a 650 nm longpass filter. Large signal peaks exceeding 200% of the mean intensity when averaged over 20 ms or more were removed from the raw data, from which the two auto- and the cross-correlation curves were computed. Samples were measured in LabTek chambered coverglasses for 240–480 s for each of four different mix preparations. For further processing and evaluation of the resulting correlation functions see Supplementary Material S1.

## Supporting Information

Supplemental Material S1 (0.17 MB DOC)

Figure S1 EM image of GTP-Tubulin oligomers EM image obtained with 25 µM GTP-tubulin in BRB80. Scale bar is 100 nm.
(7.20 MB TIF)

Figure S2 Oligomers formed by Cy3 and Cy5 labeled tubulin Concentration is 250 nM for each species. Scale bar is 100 nm.
(6.49 MB TIF)

Figure S3 Logarithm of GDP-oligomers length distribution Fitting of the experimental results (blue points) to the model (red line).
(0.13 MB TIF)

Figure S4 Image analysis of tubulin oligomers Left: A typical image on which image analysis was performed. Scale bar is 100 nm. Right: The same image after image processing as described in experimental procedures.
(20.88 MB TIF)
