# Supplementary material for: Tubulin Dimers Oligomerize before Their Incorporation into Microtubules
Source: PLoS One. 2008 Nov 27;3(11):e3821. doi: 10.1371/journal.pone.0003821 (PMC2584370; doi:10.1371/journal.pone.0003821)
Supplement: Supplemental Material S1 — (0.17 MB DOC) [file pone.0003821.s001.doc]

**Supplementary material**

**FC(C)S analysis**

The measured autocorrelation curves were fitted with the standard model function for free diffusion of several components [1], yielding the diffusion correlation times $\tau_{\text{diff},i}\propto1/D_{i}$ where the index *i* identifies the different components. Thus, the ratio of diffusion correlation times yields the ratio of diffusion coefficients. The fit of a single component to the autocorrelation curve of free Cy5 fluorophores together with the published diffusion coefficient of Cy5, *D*_Cy5_ = 280 µm^2^ s^-1^ [2] in water at room temperature, served as a reference. The curves of Cy5-labeled tubulin at 500 nM concentration were fitted with two components, one assigned to free fluorophore present in the solution, the other assigned to tubulin, for which we obtained *D*_tubulin_ = 23.4±2.2 µm^2^ s^-1^ This was then compared to the diffusion coefficient of Cy5-labeled tubulin obtained at 25 nM, *D*_tubulin,free_ = 47.5±4.8 µm^2^ s^-1^, which was assigned to non-oligomerized tubulin dimers and which was in good agreement with the published value of *D*_tubulin,free_ = 45 µm^2^ s^-1^ [3].

In the case of longitudinal oligomerization, the ratio of diffusion coefficients of free and oligomerized tubulin dimers, *D*_tubulin,free_ and *D*_tubulin_, relates to *k*, the average number of dimers per oligomer, according to [3]:

$k\approx\left(\frac{D_{\text{tubulin,free}}}{D_{\text{tubulin}}}\right)^{1.72}$ (S1)

However, the probability of an incorporated dimer to be labeled with Cy5 is 50% and the number of Cy5-labelled dimers in an oligomer obeys a binomial distribution. Since in FCS, the contribution of a species to the resulting autocorrelation function is weighted with the square of its molecular brightness, the contribution of an oligomer containing *i* dimers is weighted with the second moment of the binomial distribution and is approximately proportional to *i*^1.68^ (in contrast to *i*^2^ for the case that all dimers carry a Cy5 label). Thus, the apparent number of dimers per oligomer as observed with FCS is

$k=\int di i^{1.68} \psi\left(i\right) \text{with} \psi\left(i\right)=\exp\left(-i/\left\langle i\right\rangle\right)/\left\langle i\right\rangle$ (S2)

where $\psi\left(i\right)$ is the exponential probability distribution of the number of *i*-mers. Executing the integration results in a corrected number of dimers per oligomer *k*_corr_:

$k=\Gamma\left(2.68\right)\left\langle i\right\rangle^{1.68}\approx1.52\left\langle i\right\rangle^{1.68} \Rightarrow k_{\text{corr}}=\left\langle i\right\rangle\approx\left(k/1.52\right)^{0.60}$ (S3)

A good observable for the amount of cross-correlation, which reflects the amount of oligomerization of differently labeled equimolar constituents, is the cross-correlation function normalized to the geometric average of the autocorrelation amplitudes [1] and more compact the normalized cross-correlation amplitude

$\text{ratioG}=\frac{G_{\text{cross}}\left(0\right)}{\sqrt{G_{\text{auto},1}\left(0\right)G_{\text{auto},2}\left(0\right)}}$ (S4)

It represents the correlation coefficient of the signal fluctuations in the two channels, which is zero in the absence of interactions and equal to one when only double-labeled particles are present. However, cross-talk results in a baseline for non-interacting particles, which was determined using the Cy3/Cy5 mix. The cross-correlation exceeds the baseline only in the presence of complexes carrying both labels, i.e. tubulin dimers in an oligomeric state.

**Analytical calculation of oligomer size distribution**

This case is known as the isodesmic assembly and has been previously described [4]. We consider the polymerization reaction:

$\text{A}+\text{A}_{i}\leftrightarrow\text{A}_{i+1}$ (S5)

The equilibrium constant is:

$K_{\text{eq}}=\frac{\left[\text{A}_{i+1}\right]}{\left[\text{A}\right]\left[\text{A}_{i}\right]}$ (S6)

The equilibrium constant is linked to *E*_long_, the free energy of longitudinal interaction:

$K_{\text{eq}}=e^{-\frac{E_{\text{long}}}{kT}}$ (S7)

This leads to an exponential distribution for the concentration *n* of oligomers of size *i*:

$n\left(i\right)=\left[\text{A}_{i}\right]=Ae^{-\frac{i-1}{i_{0}}}$ (S8)

From the normalization condition, where *φ* is the total tubulin concentration,

$\sum_{i=1}^{\infty} i\cdot n\left(i\right)=\phi$ (S9)

we can write the full expression for *n*

$n\left(i\right)=\phi\cdot\left(1-e^{-\frac{1}{i_{0}}}\right)^{2}\cdot e^{-\frac{i-1}{i_{0}}}$ (S10)

From equations (S6), (S7) and (S10), we can then write *i*_0_ as a function of the longitudinal free energy of interaction *E*_long_

$\frac{e^{-\frac{1}{i_{0}}}}{\left(1-e^{-\frac{1}{i_{0}}}\right)^{2}}=\phi\cdot e^{-\frac{E_{\text{long}}}{kT}}$ (S11)

**Effect of crowding on oligomer size distribution**

To generate a rough estimate of the macromolecular crowding effect on tubulin oligomerization, we used the results reported by Snoussi and Halle [5] on BPTI oligomerization in a Dextran solution. We considered the volume fraction occupied by macromolecules in the cytoplasm to be 0.2 and used figure 6 of Snoussi and Halle [5] to get an estimation of Γ, the factor relating the standard association constant with the association constant in the presence of a crowding agent. As they described the formation of decamers, we took the ninth root of the value they find for Γ. This gives us a decrease in the E_long_ of around 1.7 kT, leading to the formation of longer oligomers.

**References**

1. Strohner R, Wachsmuth M, Dachauer K, Mazurkiewicz J, Hochstatter J, et al. (2005) A 'loop recapture' mechanism for ACF-dependent nucleosome remodeling. Nat Struct Mol Biol 12: 683-690.

2. Bark N, Foldes-Papp Z, Rigler R (1999) The incipient stage in thrombin-induced fibrin polymerization detected by FCS at the single molecule level. Biochem Biophys Res Commun 260: 35-41.

3. Krouglova T, Vercammen J, Engelborghs Y (2004) Correct diffusion coefficients of proteins in fluorescence correlation spectroscopy. Application to tubulin oligomers induced by Mg2+ and Paclitaxel. Biophys J 87: 2635-2646.

4. Oosawa F, Asakura S (1975) Thermodynamics of the Polymerization of Proteins. New York: Academic Press.

5. Snoussi K, Halle B (2005) Protein self-association induced by macromolecular crowding: a quantitative analysis by magnetic relaxation dispersion. Biophys J 88: 2855-2866.

**Supplementary figure legends**

**Figure S1.** **EM image of GTP-Tubulin oligomers**

EM image obtained with 25 µM GTP-tubulin in BRB80. Scale bar is 100 nm.

**Figure S2. Oligomers formed by Cy3 and Cy5 labeled tubulin**

Concentration is 250 nM for each species. Scale bar is 100 nm.

**Figure S3. Logarithm of GDP-oligomers length distribution**

Fitting of the experimental results (blue points) to the model (red line).

**Figure S4. Image** **analysis of tubulin oligomers**

Left: A typical image on which image analysis was performed. Scale bar is 100 nm.

Right: The same image after image processing as described in experimental procedures.
