# Supplementary figures and images for: Tubulin Dimers Oligomerize before Their Incorporation into Microtubules
Source: PLoS One. 2008 Nov 27;3(11):e3821. doi: 10.1371/journal.pone.0003821 (PMC2584370; doi:10.1371/journal.pone.0003821)

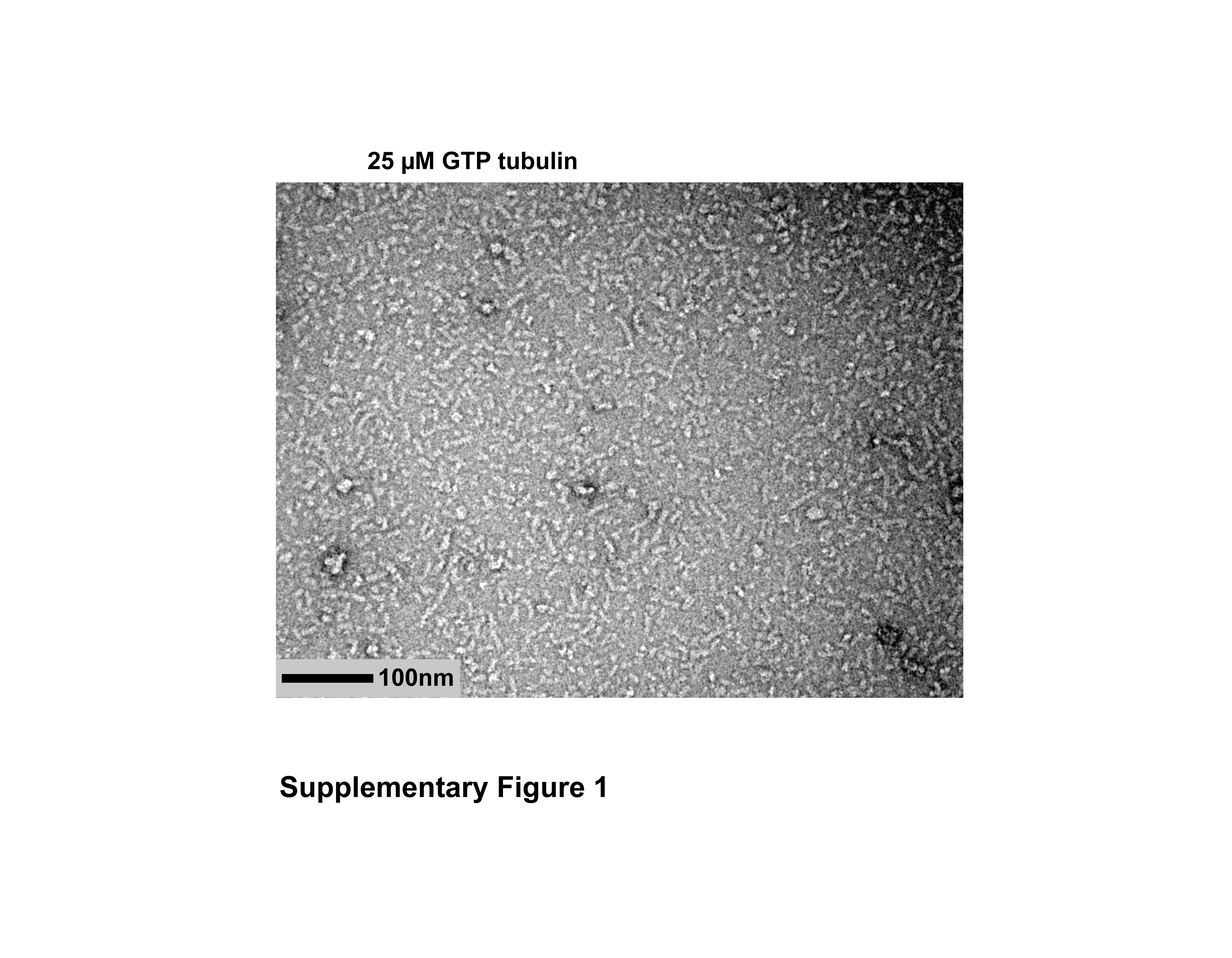

Supplement: Figure S1 — EM image of GTP-Tubulin oligomers EM image obtained with 25 µM GTP-tubulin in BRB80. Scale bar is 100 nm. (7.20 MB TIF) [file pone.0003821.s002.tif]

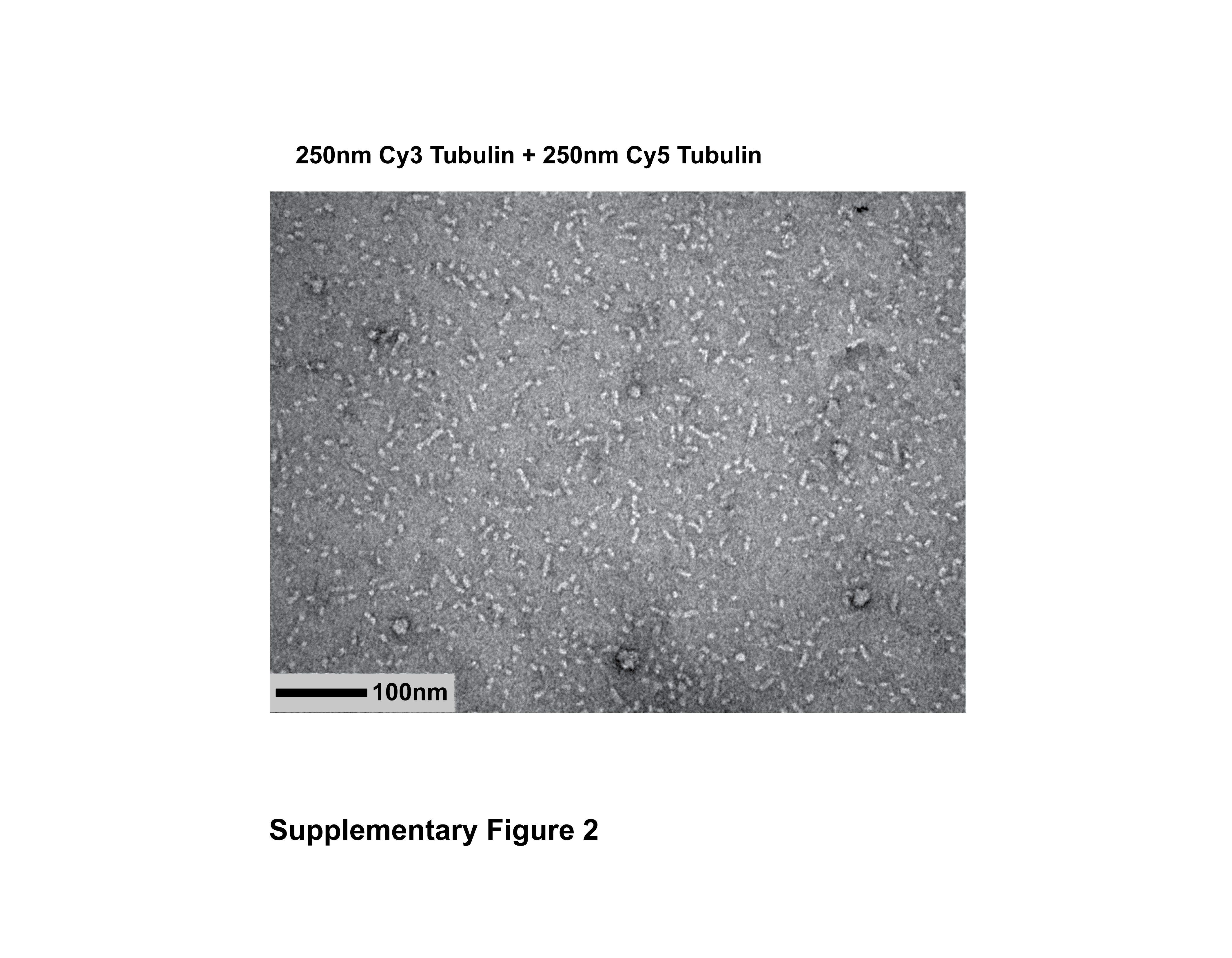

Supplement: Figure S2 — Oligomers formed by Cy3 and Cy5 labeled tubulin Concentration is 250 nM for each species. Scale bar is 100 nm. (6.49 MB TIF) [file pone.0003821.s003.tif]

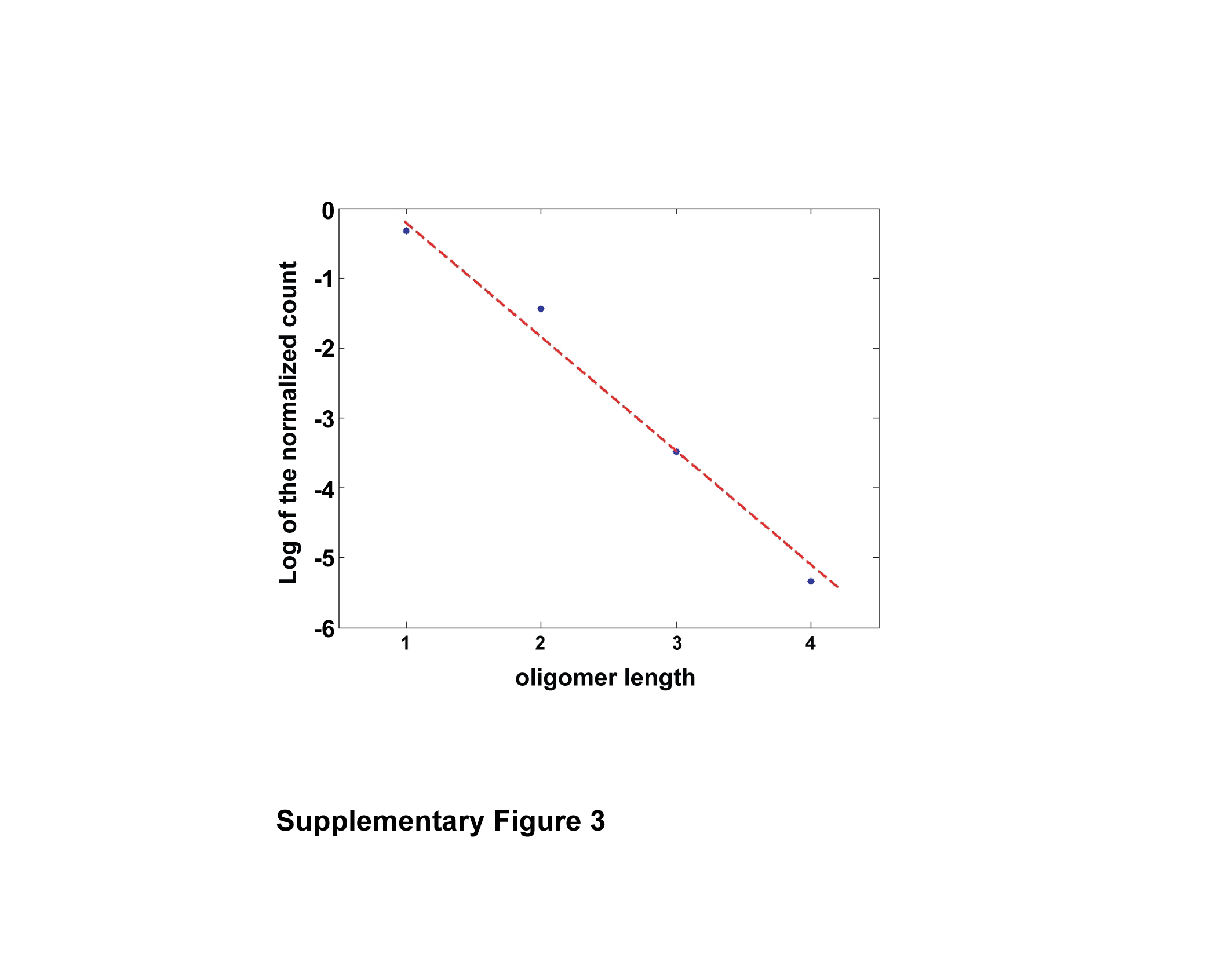

Supplement: Figure S3 — Logarithm of GDP-oligomers length distribution Fitting of the experimental results (blue points) to the model (red line). (0.13 MB TIF) [file pone.0003821.s004.tif]

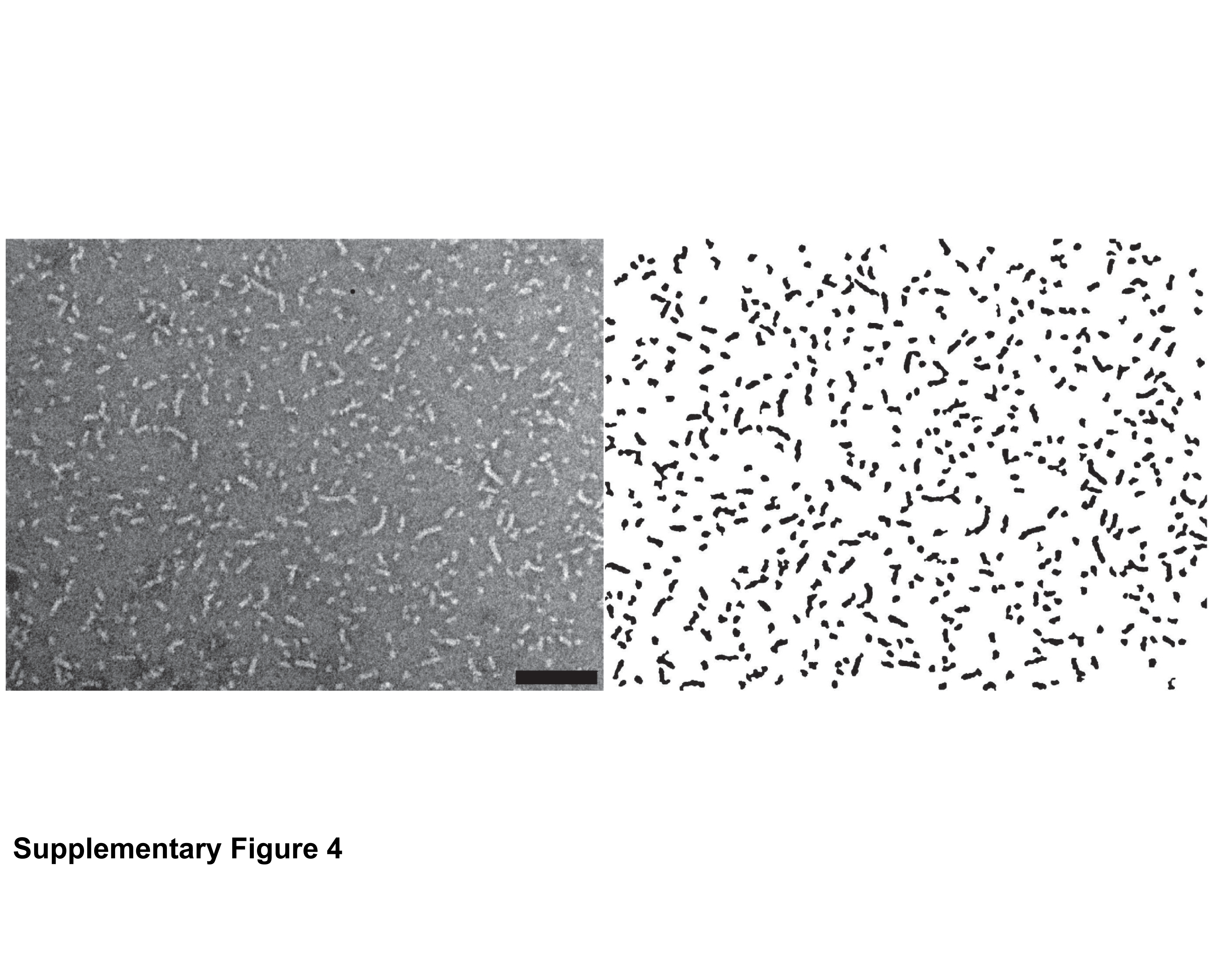

Supplement: Figure S4 — Image analysis of tubulin oligomers Left: A typical image on which image analysis was performed. Scale bar is 100 nm. Right: The same image after image processing as described in experimental procedures. (20.88 MB TIF) [file pone.0003821.s005.tif]
